# Cerebellar Leukoencephalopathy following Fentanyl Intoxication. A case report and review of the literature

**DOI:** 10.5339/qmj.2023.36

**Published:** 2024-01-06

**Authors:** Hamzah Adel Ramawad, Amirmohammad Toloui, Victor Chen, Yan Sun

**Affiliations:** 1 NYC Health & Hospital, South Brooklyn Health, Brooklyn, New York, USA Email: hamzahr.adel@gmail.com ORCID iD: 0000-0002-9687-3599; 2 Iran University of Medical Science, Tehran, Iran; 3 New York Institute of Technology College of Osteopathic Medicine, New York, USA Email:hamzahr.adel@gmail.com ORCID iD: 0000-0002-9687-3599

**Keywords:** Fentanyl overdose, opioid, addiction medicine, ataxia, leukoencephalopathy, altered mental status

## Abstract

The opioid crisis has become a significant public health concern in recent years. Although respiratory depression and overdose are the most reported side effects of fentanyl, there have been rare cases of cerebellar leukoencephalopathy (CLE) following fentanyl intoxication. A 29-year-old man with a history of opioid use disorder and intravenous drug use presented to the emergency room with significant ataxia and dysarthria following fentanyl intoxication. According to the patient, the symptoms began four days prior after “chasing the dragon” with “pure fentanyl”, and he reported experiencing nausea and dizziness, particularly during ambulation. Neurological examination revealed a positive Romberg test, ataxia, and delayed speech. Brain magnetic resonance imaging (MRI) indicated there was toxic degeneration of the cerebellar white matter that extended into the posterior limbs of the internal capsule. Urine drug screening was positive for opioids, making fentanyl-induced cerebellar leukoencephalopathy the most likely diagnosis in this case. This case of opioid-induced CLE underscores the critical significance of early recognition, which is vital for enhancing a patient’s recovery and averting the development of severe neurological complications.

## Introduction

The opioid crisis has become a significant public health concern in recent years, with synthetic opioids such as fentanyl being responsible for a large number of overdose deaths in the United States. In 2020, the Centers for Disease Control and Prevention (CDC) recorded over 42,687 overdose deaths linked to fentanyl.^1^ Although respiratory depression and overdose are the most reported side effects of fentanyl, there have been rare cases of cerebellar leukoencephalopathy (CLE) following fentanyl intoxication.^2,3^ CLE is a rare disorder characterized by inflammation and demyelination of the cerebellar white matter, leading to ataxia, nystagmus, and dysarthria.^4^ Involved patients often present with decreased levels of consciousness, vomiting, ataxia, aphasia, and hypotonia. MRI is the method of choice for determining cerebellar involvement since the precise pathophysiology of this condition remains unknown.^5^ Here, we present the case of a 29-year-old male who experienced CLE and cerebellar ataxia following fentanyl intoxication, with prominent cerebellar lesions evident on neuroimaging.

## Case Report

**Figure 1.**
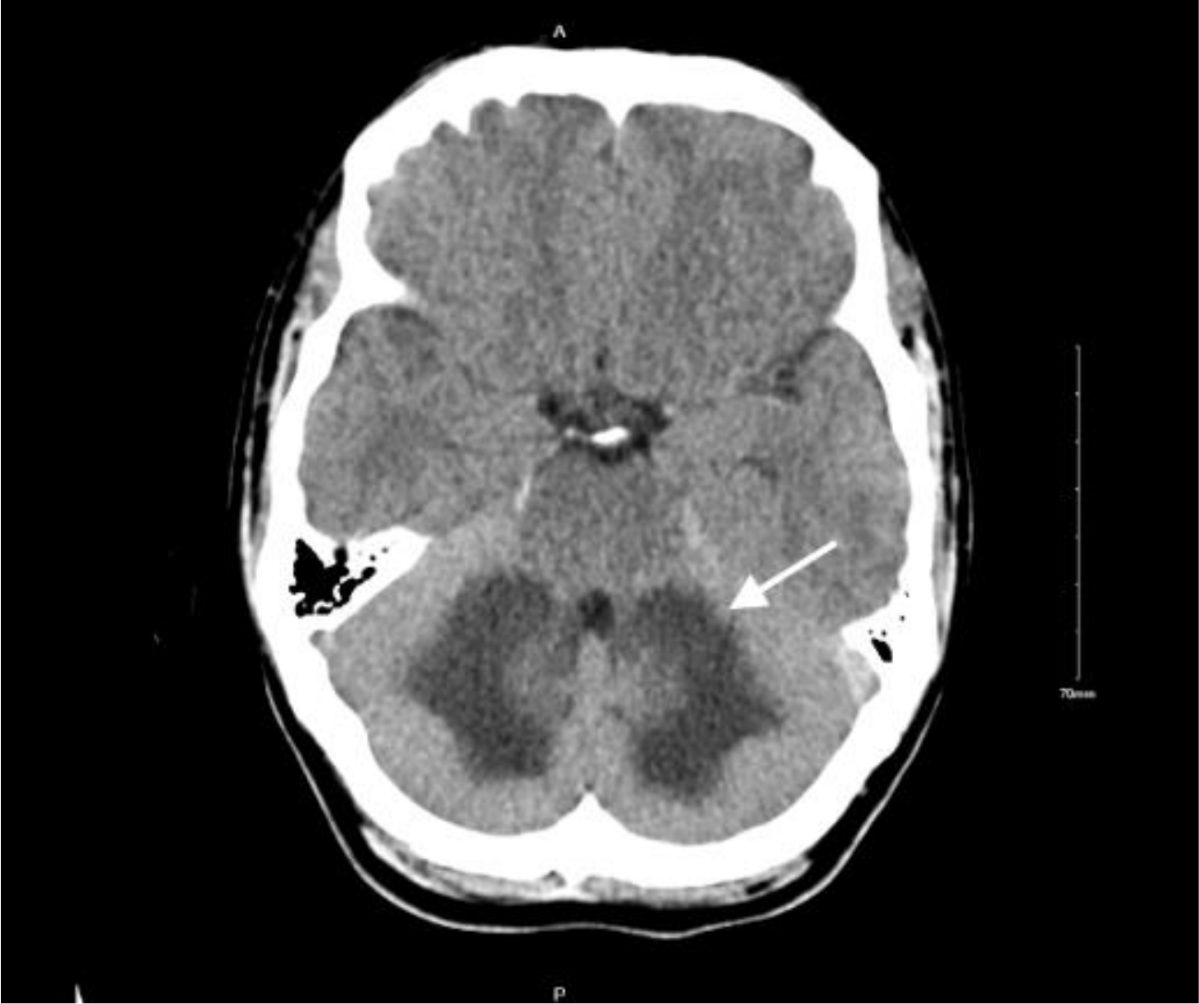
Axial head computed tomography (CT). Symmetric low attenuation lesions involving bilateral cerebellum measuring 5cm by 3cm are suspicious for acute to subacute infarct.

**Figure 2.**
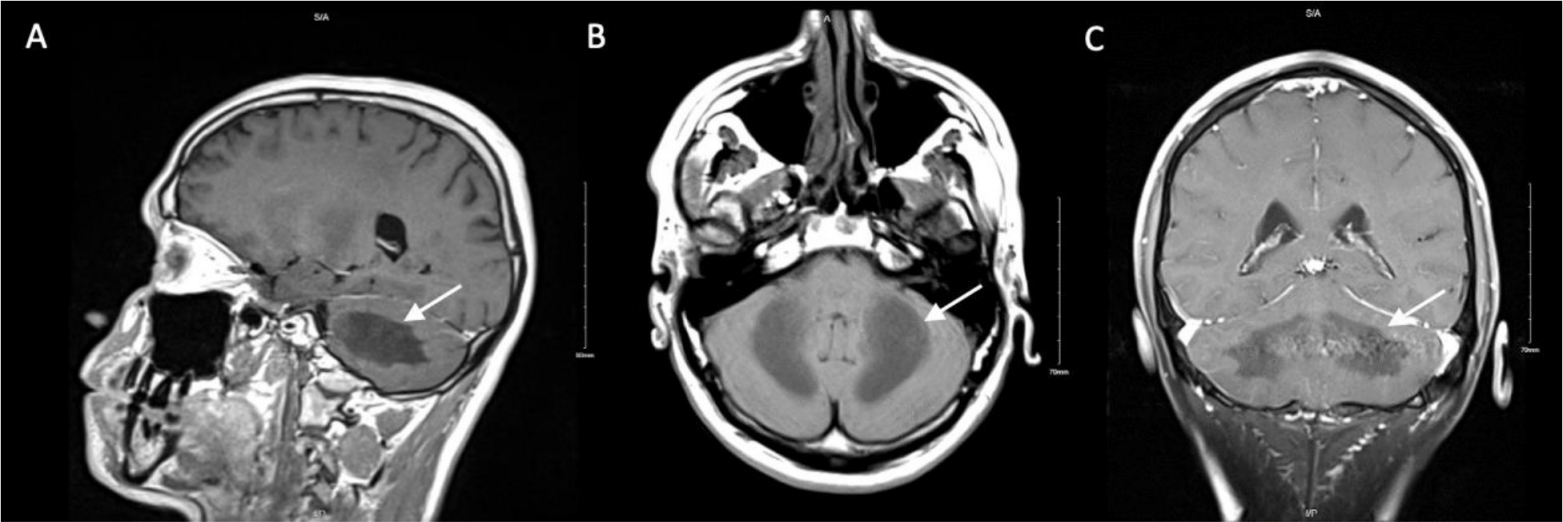
Brain magnetic resonance imaging (MRI). (A) Sagittal (B) Axial (C) Coronal images demonstrate symmetric abnormal degeneration in the cerebellar white matter extending to the posterior limbs of the internal capsule. Differential diagnosis includes acquired toxic/metabolic degenerative disorder.

A 29-year-old man with a history of opioid use disorder and intravenous drug use (IVDU) presented to the emergency room with significant ataxia and dysarthria following fentanyl intoxication. According to the patient, the symptoms began four days prior after “chasing the dragon” with “pure fentanyl”, and he reported experiencing nausea and dizziness, particularly during ambulation. On arrival at the emergency department, the patient was awake. His temperature was 98.1 °F, heart rate 93 beats/min, blood pressure 141/91 mmHg, respiratory rate 20 breaths/min, and oxygen saturation 98% on room air. Neurological examination revealed a positive Romberg test, ataxia, and delayed speech. To rule out a stroke, a computed tomography (CT) scan of the head was performed, which was suspicious for a subacute to acute infarct to the cerebellum (Figure 1). There were no signs of hydrocephalus or herniation. A subsequent brain magnetic resonance imaging (MRI) indicated there was toxic degeneration of the cerebellar white matter that extended into the posterior limbs of the internal capsule (Figure 2A, 2B, and 2C). Laboratory testing, which included electrolytes, complete blood count, renal function, and liver function showed no significant abnormalities. Urine drug screening was positive for opioids making fentanyl induced cerebellar leukoencephalopathy the most likely diagnosis in this case. Naloxone was not administered to prevent acute withdrawals, as the patient did not exhibit respiratory depression or oxygenation difficulties. After being admitted to the neurology service for further workup and evaluation, the patient remained in the hospital for seven days and showed improvements in clinical symptoms.

The in-hospital treatment for our patient involved a comprehensive approach targeting both the underlying cause, which was fentanyl intoxication, and symptom management. Supportive care included hydration and electrolyte repletion. The addiction medicine team initiated 0.1 mg of clonidine to alleviate sympathetic hyperactivity during acute opioid withdrawal. He also underwent rehabilitation, which included both physical and occupational therapies, to address his ataxia and dysarthria. These therapies ultimately helped him regain the ability to walk without difficulties and speak more clearly. Upon discharge, our patient was transitioned to methadone and was also enrolled in a methadone clinic for ongoing support and recovery efforts. Unfortunately, the patient never followed up with the neurology clinic and no further neuroimaging or progress could be obtained.

## Discussion

Fentanyl, a synthetic opioid more potent than heroin and morphine, is available in two major forms: pharmaceutical and illegally made fentanyl (IMF). The prescribed Fentanyl is widely used in the management of chronic and cancer-related pain. The IMF on the other hand, is commonly combined with other drugs, such as heroin, methamphetamine, cocaine, or other contaminants to make it cheaper and more potent. The IMF is available in the market in liquid (nasal sprays, eye drops, and edible drops), powdered, and pill forms. In the context of opioid use, it is crucial to highlight the dangerous practice of “chasing the dragon”, especially in the context of fentanyl. During this process, an individual places powdered fentanyl on a piece of aluminum foil and applies heat from below using a lighter. The resulting smoke creates a swirling effect on the foil, resembling the motion of a Chinese dragon. The user captures the smoke by inhaling the fumes through a straw or tube.^6^

Fentanyl overdose has been identified as a leading cause of opioid-related deaths in the United States.1 Although Leukoencephalopathy and ataxia are rare side effects of fentanyl intoxication, a small number of cases have documented the occurrence of these neurological complications in patients who use fentanyl.^2-4,7-9^ The molecular mechanism remains incompletely understood, with some speculating that synthetic fentanyl with potential contaminants could be a contributing factor, but the neurotoxic effects of opioids may also be involved.^10^ Opioids can induce cytotoxic effects, including secondary kidney failure due to rhabdomyolysis, direct hepatotoxicity, and an overall elevation in superoxide production, which leads to the formation of reactive oxygen species and subsequent cellular injury.^11^ While the majority of previously reported cases suggest a delayed onset of these neurological symptoms, which may be related either to the direct impact of opioid receptors on neuronal metabolism or to prolonged hypoxia, our studied patient reported the onset of symptoms right after fentanyl intoxication. Furthermore, all the previous cases in the literature involve leukoencephalopathy in the cerebrum or hippocampus in adults, whereas the neurodegenerative changes of leukoencephalopathy in our case were observed in the cerebellum, similar to those reported in the pediatric population.

The differential diagnosis of CLE resulting from fentanyl overdose is crucial to rule out other potential causes of similar neurological conditions. Toxic exposure to substances other than opioids, like certain chemotherapeutic agents, cocaine, and carbon monoxide poisoning, may lead to comparable neurological manifestations.^10^ In considering potential causes, infectious diseases such as human immunodeficiency virus (HIV) and human polyomavirus (JC virus), along with autoimmune conditions like systemic lupus erythematosus (SLE) and multiple sclerosis. Accurate diagnosis and appropriate management require a comprehensive clinical evaluation, neuroimaging, and laboratory testing. 

The diagnosis of CLE after fentanyl overdose is challenging due to the nonspecific symptoms and lack of specific diagnostic criteria. However, awareness of opioid-related neuropathology may be critical in the care of patients in the emergency department. The diagnosis is usually made based on clinical presentation and neuroimaging findings. During the neurological physical examination, specific tests are used to assess cerebellar function. These tests encompass gait and balance evaluation, pronator drift assessment, the finger-to-nose test, rapid alternating action examination, and the heel-to-shin test. In our patient’s case, a positive Romberg test was observed. This outcome typically indicates a loss of balance and stability when standing with feet together and eyes closed. Such a result can be indicative of sensory or proprioceptive dysfunction within the nervous system, potentially suggesting issues with the patient’s sense of body position and coordination, processes that are governed by the cerebellum-imaging studies including CT and MRI. However, MRI is more sensitive in detecting changes in the cerebellar white matter seen in CLE. The typical MRI finding of CLE includes diffuse T2 hyperintensity and is restricted in the cerebellar white matter.^12^ When autoimmune conditions are suspected as the potential cause of CLE, a comprehensive autoimmune evaluation should be conducted. This evaluation may include testing for nonspecific antinuclear antibodies (ANA).^13^ If there is suspicion of opioid use and a patient presents with respiratory depression or is comatose in the setting of opioid suspicions, naloxone reversal should be considered first.^14^

The management of CLE after fentanyl intoxication is primarily supportive, focusing on controlling elevated intracranial pressure and minimizing the risk of secondary neurological complications. Reported treatments include hyperosmolar therapies, corticosteroids, and vasoactive infusion to maintain cerebral perfusion.^11^ Naloxone is often administered to reverse any residual opioid effects. In severe cases where cerebellar edema can cause hydrocephalus and subsequent compression of the fourth ventricle and hydrocephalus, respiratory support such as oxygen supplementation and mechanical ventilation may be necessary. In such cases, emergency neurosurgical evaluation and intervention such as an extra ventricular drain, ventriculostomy, or suboccipital decompressive craniectomy may be lifesaving.^15^ Additionally, the presence of edema may indicate a need for intravenous methylprednisolone.^16^ However, for patients without these findings, a lumbar puncture should be performed to rule out meningitis or encephalitis. In our case, the patient has CLE with ataxia, slowing of speech, and nausea, but no cerebellar edema, compression of the ventricles, or hydrocephalus was observed. Rehabilitation therapies, such as physical and occupational therapy, can be beneficial in helping patients regain functional independence and improve their overall well-being. 

## Conclusion

CLE is a rare but potentially serious neurological complication of fentanyl overdose that emergency room doctors should consider. The diagnosis of CLE can be aided by MRI imaging. The management is mainly supportive, although severe cases may require airway protection and emergency neurosurgical intervention. With the rise in illicit fentanyl use, emergency department doctors should give more consideration to CLE and cerebellar ataxia as additional cases of neuropathology linked to opioids.

## Acknowledgments

We extend our gratitude to the healthcare professionals in the emergency department at South Brooklyn Health who were actively involved in providing care for this patient. 

## Ethical Approval

This case report received ethical approval from the New York City Health and Hospitals

Academic Committee at South Brooklyn Health (Confirmation Code: CIH-07-23). We conducted this study per the Helsinki Declaration as revised in 2013.

## Disclosures

Consent: Verbal and written consent for publication was obtained from the patient.

Sources of funding: This work did not receive funding.

Conflict of interest: All authors have declared that they have no conflict of interest.

## Authors Contribution

Study design: All authors.

Data gathering: All authors.

Data analysis: N/A.

Interpreting the findings: All authors.

Manuscript writing: All authors.
